# Combined EGFR and VEGFR versus Single EGFR Signaling Pathways Inhibition Therapy for NSCLC: A Systematic Review and Meta-Analysis

**DOI:** 10.1371/journal.pone.0040178

**Published:** 2012-08-16

**Authors:** Xinji Zhang, Yesheng Li, Hui Li, Yingyi Qin, Chong Bai, Feng Xu, Tianyi Zhu, Jinfang Xu, Mengjie Wu, Chaoxiang Wang, Lixin Wei, Jia He

**Affiliations:** 1 Department of Health Statistics, Second Military Medical University, Shanghai, China; 2 Department of Special Treatment, Eastern Hepatobiliary Hospital, Second Military Medical University, Shanghai, China; 3 Respiratory Department, Changhai Hospital, Second Military Medical University, Shanghai, China; 4 Tumor Immunology and Gene Therapy Center, Eastern Hepatobiliary Hospital, Second Military Medical University, Shanghai, China; National Taiwan University Hospital, Taiwan

## Abstract

**Background:**

Lung cancer is a heterogeneous disease with multiple signaling pathways influencing tumor cell survival and proliferation, and it is likely that blocking only one of these pathways allows others to act as salvage or escape mechanisms for cancer cells. Whether combined inhibition therapy has greater anti-tumor activity than single inhibition therapy is a matter of debate. Hence, a meta-analysis comparing therapy inhibiting both VEGFR and EGFR signaling pathways with that inhibiting EGFR signaling pathway alone was performed.

**Methodology and Principal Findings:**

We searched PubMed, EMBASE database and the proceedings of major conferences for relevant clinical trials. Outcomes analyzed were objective tumor response rate (ORR), progression-free survival (PFS), overall survival (OS) and toxicity. Besides, subgroup analyses were performed to investigate whether the combined inhibition therapy is best performed using combination of selective agents or a single agent with multiple targets.

Six trials recruiting 3,302 patients were included in the analysis. Combined inhibition therapy was associated with a 3% improvement in OS as compared with single-targeted therapy, but this difference was not statistically significant (HR, 0.97; 95% CI, 0.89–1.05; P = 0.472). Patients receiving combined inhibition therapy had significant longer PFS than the group with single-targeted therapy (HR, 0.80; 95% CI, 0.67–0.95; P = 0.011). There was no difference in the ORR between the groups (OR, 1.44; 95% CI, 0.95–2.18; P = 0.085). Subgroup analysis revealed that combined inhibition therapy using combination regimens was associated with statistically significant improvement in both ORR and PFS. Toxicity was greater in combined inhibition therapy.

**Conclusions:**

There is no evidence to support the use of combined inhibition therapy in unselected patients with advanced NSCLC. However, given the significant advantage in ORR and PFS, combined inhibition therapy using combination regimens may be considered for further evaluation in subsets of patients who may benefit from this treatment.

## Introduction

Non-small-cell lung cancer (NSCLC) accounts for approximately 80–85% of all cases of lung cancer, and is the most common cause of cancer death in industrialized countries [1]. With the notion that a “efficacy plateau” has been achieved with traditional cytotoxic chemotherapy, the treatment armamentarium for advanced NSCLC has expanded to include molecular targeted therapies that act specifically against key components of cellular pathways involved in tumor growth, progression, and cell death. Vascular endothelial growth factor (VEGF) and epidermal growth factor receptor (EGFR) inhibitors are two key molecular targeted therapies in NSCLC. Vascular endothelial growth factor (VEGF or VEGFA) is a key circulating proangiogenic factor which binds to receptors present on endothelial cells (mainly VEGFR2) [2], [3]. VEGF binding induces receptor dimerization and results in autophosphorylation which promotes binding of a number of signaling molecules and activation of intracellular signaling pathways pivotal to the process of angiogenesis [4]. In the pathologic state, VEGF production is increased by tumor cells, which stimulates the endothelial cells in existing vessels to promote the production of new vasculature via direct stimulation of signaling pathways and induction of downstream gene expression [5]. The EGFR is a receptor tyrosine kinase (TK) of the ErbB/HER family. It is expressed at high levels on the surface of many epithelial tumours, including NSCLC and is activated by a variety of ligands principally transforming growth factor alpha and epidermal growth factor [6]. Ligand binding to EGFR induces receptor homo- or hetero-dimerization and results in the activation of an intracellular tyrosine kinase domain. Receptor activation signals key downstream pathways that regulate cell proliferation, differentiation, and survival [7]. Given their prominent role in tumour growth, invasion, and metastasis, the VEGFR and EGFR signaling pathway present feasible targets for pharmacologic intervention in NSCLC, and several agents have demonstrated encouraging antitumor activity. The addition of bevacizumab, a monoclonal antibody against VEGF, to paclitaxel and carboplatin provided clinical benefit in previously untreated non-squamous advanced NSCLC [8]. And the small–molecule EGFR inhibitors, gefitinib and erlotinib, has both demonstrated anti-tumor activity in the treatment of advanced NSCLC [9]–[11].

Despite all of these improvements, the benefits associated with these agents are modest and serve to stress the need for novel therapeutic approaches. Increasing evidence has suggested that solid tumors have multiple salvage and resistance pathways that allow them to circumvent inhibition of a single signaling pathway [12]. Furthermore, NSCLC is a heterogeneous disease and it is believed that there is multi-level cross-stimulation among targets along several pathways of signal transduction that lead to tumor malignancy [13]. In fact, EGFR is known to regulate the production of VEGF and other proangiogenic factors [14], and increased VEGF expression has been associated with resistance to EGFR inhibition in a human tumor xenograft model of NSCLC [15]. Thus, it is likely that blocking only one of these pathways will be insufficient for providing any meaningful therapeutic outcomes. Therefore, a logical strategy for improving anti-tumor efficacy is inhibition of both VEGFR and EGFR signaling pathways, which may help increase suppression of oncogenic processes involved in disease progression [5], [16]–[18]. Actually, several preclinical studies have showed an enhanced benefit from combination EGFR and VEGFR inhibitors in lung cancer cell lines [19], [20]. And combined blocking of VEGFR and EGFR signaling was found to have the potential to overcome primary or acquired resistance to EGFR inhibitors in xenograft models [15], [21].

However, several randomized trials [22]–[27] comparing therapy inhibiting both VEGFR and EGFR signaling pathways with that inhibiting EGFR signaling pathways alone have been conducted and the results were various. Uncertainty remains regarding the presence and magnitude of any improvement in anticancer efficacy of the strategy of combined inhibition of the VEGFR and EGFR signaling pathways for advanced NSCLC. And we were unable to locate any meta-analyses that analyzed and summarized the evidence on the combined inhibition therapy. Hence, we performed a systematic review and meta-analysis of randomized controlled trials to evaluate the effects of the strategy of combined inhibition of the VEGFR and EGFR signaling pathways on overall survival, progression-free survival, response rate and toxicity in patients with advanced NSCLC.

## Methods

### Search strategy and selection criteria

For inclusion in this meta-analysis, randomized controlled trials were required to compare therapy inhibiting both VEGFR and EGFR signaling pathways with that inhibiting EGFR signaling pathway alone in the treatment of patients with stage IIIB or IV NSCLC. Approach to inhibiting both VEGFR and EGFR signaling pathways could be a single agent with multiple targets or a combination of targeted agents. Trials comparing the combined inhibition therapy with a combination of an agent targeting EGFR signaling pathway and a cytotoxic chemotherapy were not eligible.

Relevant studies were identified by searching PubMed, and EMBASE up to Nov 2011 without language restrictions. We performed the search by using the terms “NSCLC,” “non-small-cell lung cancer,” “carcinoma and non-small-cell lung,” “VEGFR,” “EGFR,” “clinical trial,” and “randomized trial.” This search was supplemented by a manual search the annual meeting proceedings of American Society of Clinical Oncology (ASCO) and European Society of Medical Oncology (ESMO) from 2004 to 2011. The relevant reviews and meta-analyses regarding the role of combined inhibition therapy for NSCLC patients were examined for potential inclusive trials. Moreover, we also searched in http://www.who.int/triasearch and http://www.ClinicalTrials.gov websites for information on registered randomized controlled trials.

### Data extraction and quality assessment

Data abstraction and quality assessment were conducted independently by 2 reviewers using a standardized approach. Disagreements were adjudicated by a third reviewer after referring to the original articles.

Data retrieved from the reports included publication details, methodological components, and trial characteristics such as sample size, interventions, and outcome measures. End points of interest included overall survival (OS), progression-free survival (PFS), objective tumor response rate (ORR) and adverse events (AEs). The quantitative 5-point Jadad scale [28] was used to assess the quality of the inclusive trials based on the reporting of the studies' methods and results.

### Statistical analysis

For time-to-event data, the log hazard ratios (HRs) and their variances were estimated using the methods proposed by Parmar [29] when confidence intervals (CIs) of HRs were reported. The summary HRs and their 95% CIs were estimated using a general variance-based method.

For objective tumor response rate (ORR) and toxicities, estimates of the treatment effects were obtained from the number of events reported in each arm and combined using the methods reported by Mantel and Haenszel [30]. To calculate ORR, patients obtaining complete response or partial response were considered as responders. The AEs of treatments were analyzed as drug-related WHO grades 3 or greater toxicity. An odd ratio (OR) >1 indicates a higher tumor response rate and more toxicity in the combined inhibition arm.

The χ2 test and I^2^ statistic were employed to assess variability across studies attributable to heterogeneity beyond chance [31]. A p-value greater than 0.10 for the χ2 test and an I^2^ value less than 25% were interpreted as signifying low-level heterogeneity. When there was no statistically significant heterogeneity, a pooled effect was calculated with a fixed-effect model; otherwise, a random-effect model was employed. Subgroup analyses were performed to determine if the results were influenced by different approaches to inhibiting both VEGFR and EGFR signaling pathways (a single agent or a combination of agents). We also assessed the probability of publication bias with Egger's test [32] and Begg-Mazumdar test [33]. Statistical significance was defined as a two-tailed p-value less than 0.05. All statistical analyses were conducted with the software Stata 11.0.

## Results

### Trial characteristic

**Figure 1:**
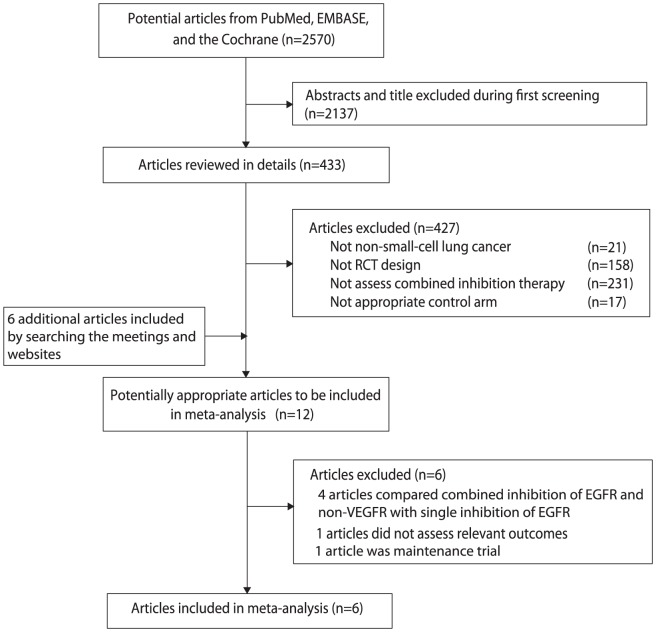
Identification process for eligible studies.

Our systematic search identified 2570 potentially relevant abstracts, of which 12 potentially eligible trials that had investigated combined inhibition therapy versus single inhibition therapy were identified (Figure 1). Of these, six trials were excluded: 4 trials [34]–[37] compared combined inhibition of EGFR and non-VEGFR signaling pathways (c-MET, HDAC, mTOR) with single inhibition of EGFR signaling pathway; 1 trial [38] was maintenance trial and 1 trial [39] did not assess relevant outcomes.

Finally, 6 trials including 3,302 patients that met the inclusion criteria were included in the meta-analysis. Four trials [22]–[24], [27] were published in full articles, while two [25], [26] were published only as meeting abstracts. All trials included patients with stage IIIB to IV NSCLC. 2 trials [22], [23] assessed vandetanib, a single multi-targeted agent inhibiting both VEGFR and EGFR signaling pathways, whereas the other 4 trials [24]–[27] assessed a combination of targeted agents as combined inhibition therapy. Table 1 summarized the characteristics of the 6 included trials.

**Table 1:** Characteristics of included trials.

Authors (Year)	Number of Patients	Therapy of Treatment And Control Arm	Male (%)	Median Age (years)	Stage IV (%)	WHO PS = 2 (%)	Jadad Score
Ronald B. Natale et al. (2009) [22]	168	Vandetanib 300 mg once-daily until PD or PT	48 (58)	63	69 (83)	0 (0)	4
		Gefitinib 250 mg once-daily until PD or PT	52 (61)	61	63 (74)	0 (0)	
H.J.M.Groen et al. (2010) [25]	132	Sunitinib 37.5 mg once daily plus Erlotinib 150 mg once daily until PD or PT	39 (60)	NR	NR	NR	4
		Placebo plus Erlotinib 150 mg once daily until PD or PT	45 (67)	NR	NR	NR	
Ronald B. Natale, et al. (2011) [23]	1240	Vandetanib 300 mg/d until PD or PT	381 (61)	61	517 (83)	65 (10)	4
		Erlotinib 150 mg/d until PD or PT	393 (64)	61	519 (84)	77 (13)	
Roy S.Herbst et al. (2011) [24]	636	Bevacizumab 15 mg/kg on the first day of 3-week cycles (±4 days) plus Erlotinib 150 mg/day until PD or PT	171 (54)	65	NR	23 (7)	5
		Placebo 15 mg/kg on the first day of 3-week cycles (±4 days) plus Erlotinib 150 mg/day until PD or PT	170 (54)	65	NR	20 (6)	
David R.Spigel et al. (2011) [27]	166	Sorafenib 400 mg orally twice a day plus Erlotinib 150 mg orally daily until PD or PT	62 (56)	65	NR	13(12)	4
		Placebo plus Erlotinib 150 mg orally daily until PD or PT	26 (47)	65	NR	10(18)	
R. Govindan et al. (2011) [26]	960	Sunitinib 37.5 mg once daily plus Erlotinib 150 mg once daily until PD or PT	297 (62)	61	438 (91)	2 (0.4)	4
		lacebo plus Erlotinib 150 mg once daily until PD or PT	284 (59)	61	448 (93)	1 (0.2)	

Abbreviations: NR, not reported; PD, disease progression; PT, prohibitive toxicity.

Jadad scale was used to assess the quality of the included trials. Overall, one trial [24] had a Jadad score of 5, and five [22], [23], [25]–[27] scored 4.

### Overall Survival (OS)

**Figure 2:**
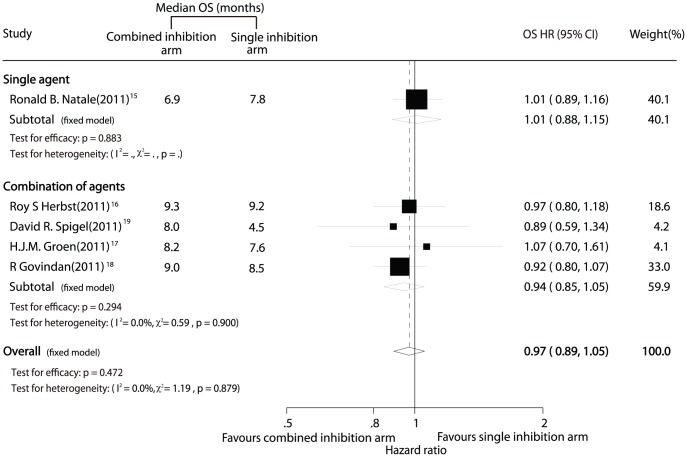
Comparison of overall survival between combined inhibition therapy and single inhibition therapy.

Data for OS were available from 5 trials [23]–[27] including 3,134 patients. The data of the trial by Natale et al. [22] was excluded from the analysis of OS in our meta-analysis because the two-part crossover design might confound assessment of the effect of vandetanib on OS. Combined inhibition therapy was associated with a 3% improvement in OS as compared with single inhibition therapy, but this difference was not statistically significant (HR, 0.97; 95% CI, 0.89–1.05; P = 0.472; Figure 2). There was no significant heterogeneity for OS among the individual trials (P = 0.88; I^2^ = 0.0%), and no evidence of significant publication bias was detected (Egger test, P = 0.956; Begg-Mazumdar test, P = 1.000).

Results were similar when subgroup analyses were conducted, with no differences detected between single inhibition therapy and combined inhibition therapy with either a single agent or a combination of agents (Figure 2).

### Progression-Free Survival (PFS)

**Figure 3:**
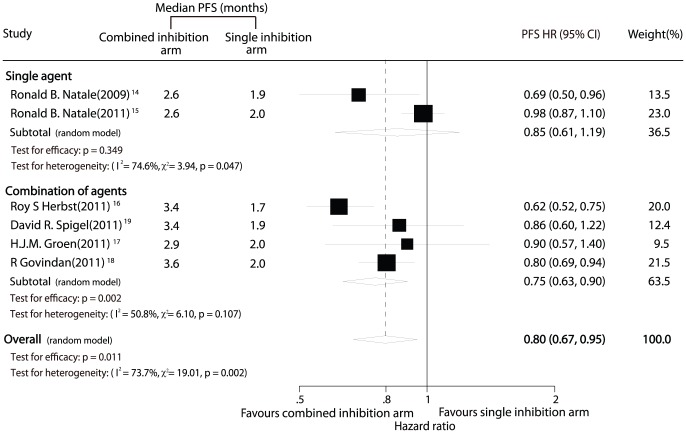
Comparison of progression-free survival between combined inhibition therapy and single inhibition therapy.

All 6 trials [22]–[27] including 3,302 patients provided PFS results. The meta-analysis revealed that combined inhibition therapy yielded a clinically and statistically significant 21% improvement in PFS compared with single inhibition therapy (HR, 0.80; 95% CI, 0.67–0.95; P = 0.011; Figure 3).

Nevertheless, there might be substantial heterogeneity in the HRs for PFS from the individual trials (P = 0.002; I^2^ = 73.7%) and we incorporated it into random-effects model. Furthermore, subgroup analysis was conducted according to the different approaches to inhibiting both VEGFR and EGFR signaling pathways. Combined inhibition therapy using combination regimen demonstrated clinically substantial and statistically significant improvement in PFS (HR, 0.75; 95% CI, 0.63–0.90; P = 0.002, Figure 3) with much less heterogeneity ((P = 0.13; I^2^ = 47%). However, no significantly statistical differences in PFS were detected between combined inhibition therapy with a single multi-targeted agent and single inhibition therapy (HR, 0.85; 95% CI, 0.61–1.19; P = 0.349, Figure 3).

No evidence of publication bias was found using Egger test (P = 0.596) or Begg-Mazumdar test (P = 1.000).

### Objective Tumor Response Rate (ORR)

**Figure 4:**
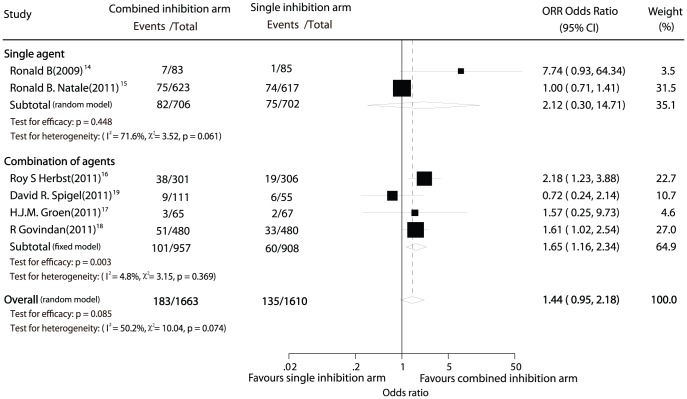
Comparison of objective tumor response rate between combined inhibition therapy and single inhibition therapy.

Data for ORR was available from 6 trials [22]–[27] including 3,273 patients. Combined inhibition therapy was associated with 11.00% absolute tumor response rate while single inhibition therapy yielded 8.39%, however, this difference was not statistically significant (OR, 1.44; 95% CI, 0.95–2.18; P = 0.085; Figure 4). There was some evidence of heterogeneity for ORR among the individual trials (P = 0.07; I^2^ = 50%). No evidence of significant publication bias was detected (Egger test, P = 0.421; Begg-Mazumdar test, P = 0.452).

In the subgroup analysis of combined inhibition therapy using a single multi-targeted agent, the result was consistent, with no significant difference in ORR between combined inhibition therapy and single inhibition therapy (OR, 2.12; 95% CI, 0.30–14.71; P = 0.448, Figure 4).

However, combined inhibition therapy using combination regimen was associated with statistically significant improvement in ORR compared with single inhibition therapy (OR, 1.66; 95% CI, 1.19–2.32; P = 0.003, Figure 4). And the effect estimate was not heterogeneous among studies (P = 0.37; I^2^ = 5%).

### Adverse events

**Figure 5:**
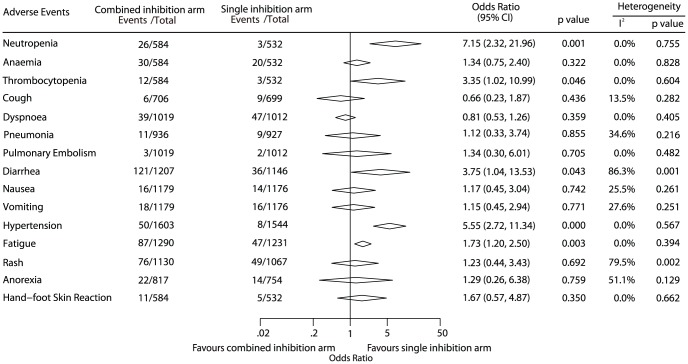
Summary of toxicities grade 3 or greater.

A summary of WHO grade 3 or greater adverse events is reported in Figure 5. Considerable variability in the completeness of toxicity reporting was found among the studies. Overall, combined inhibition therapy was associated with a significant increase in the risk for neutropenia (OR, 7.15; 95% CI, 2.32–21.96), thrombocytopenia (OR, 3.35; 95% CI, 1.02–10.99), diarrhea (OR, 3.75; 95% CI, 1.04–13.53), hypertension (OR, 5.55; 95% CI, 2.72–11.34), and fatigue (OR, 1.73; 95% CI, 1.20–2.50). Heterogeneity among individual trials was found in some adverse events analyses, possibly due to the different agents.

## Discussion

NSCLC is a heterogeneous disease and multiple signaling pathways influence tumor cell survival and proliferation. Previous studies tested the hypothesis that the therapy inhibiting both VEGFR and EGFR signaling pathways could improve survival. Although all randomised trials failed to prove gain in overall survival, some researchers advocated that new trials with bigger sample size or proper strategy would be necessary to increase overall survival. A meta-analysis of the previous publications could answer some of the questions, including if the combined inhibition therapy would really benefit the patients and which combined inhibition approach is better. This systematic review represents the best current evidence about the combined inhibition therapy in treatment of advanced NSCLC.

The pooled analysis, with data obtained from 3,302 NSCL patients, found that the treatment inhibiting both VEGFR and EGFR signaling pathways does not improve overall survival among unselected patients. Hence, existing evidence from randomized controlled trials does not support the use of combined inhibition therapy for unselected patients with advanced NSCLC. However, the subset analyses of the trial by Spigel et al. [27] suggested that sorafenib plus erlotinib was associated a statistically significant improvement in OS compared with erlotinib alone among the EGFR wild-type (WT) patients (HR, 0.53; 95% CI, 0.29–0.98; one-sided P = 0.019). Similarly, OS advantage for sorafenib plus erlotinib compared with erlotinib alone was also suggested among patients with EGFR FISH–negative cancers (median OS: 10.55 months for sorafenib plus erlotinib vs 4.60months for erlotinib; one-sided P = 0.064) [27]. And despite lack of statistical significance, results of the trial which assessed the efficacy of addition of bevacizumab to erlotinib also suggested that patients with EGFR-mutant tumours may benefit from the combined inhibition therapy (median OS: 18 months for bevacizumab plus erlotinib vs 12 months for erlotinib; HR, 0.44; 95% CI, 0.11–1.67). These suggest that combined inhibition therapy has a potential advantage in the treatment of advanced NSCLC compared with single inhibition therapy, if the subsets of patients who may benefit from this treatment are identified.

Differently, the results of this meta-analysis demonstrated that combined inhibition therapy yielded a statistically significant benefit in PFS as compared with single inhibition therapy. However, it should be noted progression-free survival was not improved when a single multi-targeted agent (vandetanib) was used to inhibit both the VEGFR and EGFR signaling pathways while delay in disease progression was observed when a combination of targeted agents was used for combined inhibition therapy. Similarly to progression-free survival, improvement in response rate was only found in combined inhibition therapy using combination regimen. One potential explanation for the negative results of combined inhibition therapy using one multi-targeted agent is its effect on inhibition both the VEGFR and EGFR signaling pathways is not as specific as that of single-targeted therapy (e.g. bevacizumab, erlotinib), which may compromise its overall anti-tumor efficacy. Although tumor biomarker analyses from the ZODIAC study [40] suggested that consistent trends toward improved OS, PFS, and objective response rate for patients with EGFR gene copy number (FISH+) or EGFR mutation status (MT) tumors were seen with vandetanib group plus docetaxel versus docetaxel alone, it is not clear whether vandetanib has a potential advantage in patients with specific biomarkers as compared with single EGFR signaling pathways inhibition therapy (e.g. erlotinib). Hence, solid recommendation of a single multi-targeted agent as combined inhibition therapy could not be given based on current evidence.

As expected, some toxicity was significantly more severe in patients who received combined inhibition therapy. Symptomatic improvement due to tumor shrinkage should be balanced with increased toxic effects of combined inhibition therapy. And Concerns remain regarding the impact of the increased toxicity of combined inhibition therapy on patients' quality of life. Unfortunately, data on quality of life were rarely available in these trials and no conclusions could be drawn. However, significant increase in some adverse events (like hypertention, diarrhea or fatigue) in the combined inhibition therapy arm may impair quality of life.

The main purpose of the meta-analysis was to present all available evidence in a systematic, quantitative, and unbiased fashion. Several technical limitations of this meta-analysis should be acknowledged. The analysis is not based on individual patient data, which might provide further insight into the efficacy of the combined inhibition therapy [41]. Heterogeneity among trials can be another limitation of our meta-analysis. We applied a random-effect model that takes possible heterogeneity into consideration and preformed subgroup analyses according to the combined inhibition approach to further explore the source of heterogeneity. It is of interest that most of the variability comes from studies using a single multi-targeted agent as combined inhibition therapy, whereas the trials using combination regimen are much more consistent with one another. Other limitations include publication status and treatment regimens.

A number of other dual-inhibition strategies (e.g. m-TOR, c-Met, IGF-1R or histone deacetylase (HDAC) inhibitor plus EGFR inhibitor) have been studied. The addition of c-Met inhibitor to erlotinib has demonstrated promising clinical activity in phase II studies [34], [42] when compared with erlotinib alone, particularly among patients with Met over expression and nonsquamous histology. MET amplification leads to EGFR-independent activation of the PI3K/Akt pathway through the activation of erbB-3-dependent signalling and thereby could lead to EGFR inhibitor resistance [43]. Thus, dual EGFR-Met inhibition has a theoretic advantage for overcoming Met-mediated resistance to EGFR inhibitors [44]. The subset analyses of the trial by Spigel et al [42] suggested that MetMab plus erlotinib were associated with increased PFS and OS as compared with erlotinib alone in patients with Met over expression. And in the study [34] comparing ARQ 197–209 plus erlotinib with erlotinib, a statistically significant improvement in OS was also found in non-squamous patients in favor of ARQ 197–209 and erlotinib combination. Again, identification of predictive markers which may enable treatments to be targeted to specific patient groups is critical.

In conclusion, the findings of this study corroborate the previous findings that the combined inhibition of the VEGFR and EGFR signaling pathways does not improve overall survival among unselected patients. However, evidences of a significant difference in PFS and ORR were found to support further study of combined inhibition therapy using combination regimen. And subgroup analyses of previous studies suggested that overall survival might be improved by sorafenib/erlotinib combination in patients with EGFR WT and EGFR FISH–negative tumors and by bevacizumab/erlotinib combination in patients with EGFR-mutant tumors. Additional study of these combinations in selected patients is warranted.
